# Primary Oral Tuberculosis of Tongue, Tonsil, and Labial Mucosa: A Rare Case Report

**DOI:** 10.1155/carm/6678465

**Published:** 2026-02-17

**Authors:** Yosief Yemane, Hailemichael Ghebremariam, Teklezghi Ainealem, Haben Daniel, Natnael Ghebregziabher

**Affiliations:** ^1^ Dekemhare Hospital, Ministry of Health, Dekemhare, Southern Zone, Eritrea, behdasht.gov.ir; ^2^ Bachelor of Science in Nursing, Dekemhare Hospital, Ministry of Health, Dekemhare, Southern Zone, Eritrea, health.go.ke

**Keywords:** case report, extrapulmonary tuberculosis, GeneXpert, tongue, tonsil, Ziehl–Neelsen

## Abstract

**Introduction:**

Tuberculosis (TB) is worldwide a leading cause of morbidity, mortality, and a major public health problem particularly in resource constrained countries. Extrapulmonary tuberculosis (EPTB) accounts for about 20% of all TB cases. Primary oral cavity TB is not only rare form of EPTB but also exists with its diagnostic challenges. To our knowledge, a small number of primary oral TB cases have been reported in literature, which is likely due to the limited diagnostic modalities and its rarity.

**Case Presentation:**

We report a 40‐year‐old female patient presented to Dekemhare hospital in Dekemhare, Southern zone, Eritrea, with ulcerated oral cavity lesions associated with painful eating and swallowing. The diagnosis of oral TB involving tonsil, tongue, and labial mucosa was confirmed with Ziehl–Neelsen method and GeneXpert MTB/RIF molecular test from pus scrubbed from the lesions. The patient was treated with current anti‐TB regimen.

**Conclusion:**

Since TB can affect any site of the body, high degree of suspicion is very important in its diagnosis. This case underscores the importance of considering TB in the differential diagnosis of chronic oral ulcers, especially in endemic regions, and highlights the need for awareness among oral health professionals to facilitate early diagnosis and management of this rare manifestation.

## 1. Introduction

Tuberculosis (TB) is a chronic communicable disease that is a major cause of ill health, ranked second‐leading cause of death from a single infectious agent after COVID‐19 in 2022 [1], ranking first above HIV/AIDS in 2019 [2]. TB is curable and preventable yet the deaths it caused was almost twofold as many deaths as HIV/AIDS in 2022 [1].

TB is caused by inhalation of the bacillus *Mycobacterium tuberculosis*, which is usually spread when infected person expel bacteria into the air [1, 3]. After inhaling the mycobacterium, a person may remain asymptomatic for years as the infection might be suppressed by the strong hostimmune system [3, 4]. Among those who become infected, about 90%–95% never develop TB disease and their infection heals but the bacilli remain dormant within the body for a long time [3] and for the remaining cases, usually the disease flares up when the protective capacity of the body is compromised. There are a number of risk factors related to the high burden of TB; the major ones being HIV infection, smoking, diabetes, alcohol use, malnutrition [1, 3], time since infection (recent infection), and poorly treated previous TB [3]. However, in a significant proportion of cases, the risk factor is unknown [3].

TB can affect any sites of the body, which is classified as pulmonary and extrapulmonary TB (EPTB). EPTB is relatively uncommon (seen in 10%–15% of the cases) and can involve multiple sites’ lymph nodes, peritoneum, genitourinary, musculoskeletal, nervous, and hepatosplenic systems, spreading either via self‐inoculation from or through blood/lymph [5]. The occurrence of oral TB is very rare and its prevalence varies from studies, generally ranges 0.05%–5% [4, 6].

Although the occurrence of oral TB is a rare entity, its consideration in a long‐standing oral cavity ulcer is important in its diagnosis and early treatment. Oral health providers need to be aware of the disease burden and enhance its identification as they are the forefront in the diagnosis of oral lesion conditions.

A case of primary oral TB without any other cardinal symptoms and signs, affecting the tongue, right palatine tonsil, and left labial mucosa commissure of the mouth in a 40‐year‐old female patient will be presented here.

## 2. Case Presentation

**Figure 1:**
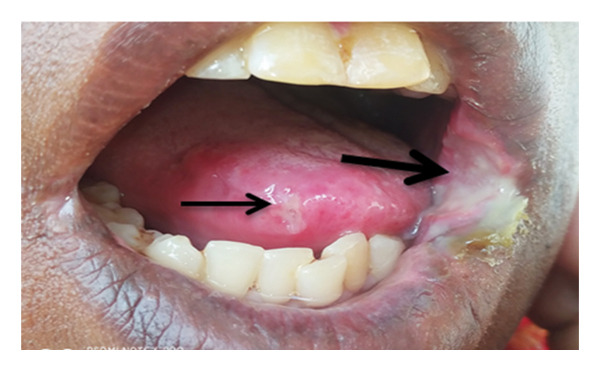
Ulcer on the tongue (thin arrow) and left labial mucosa commissure of the mouth (thick arrow) with exudates and elevated edges during presentation.

**Figure 2:**
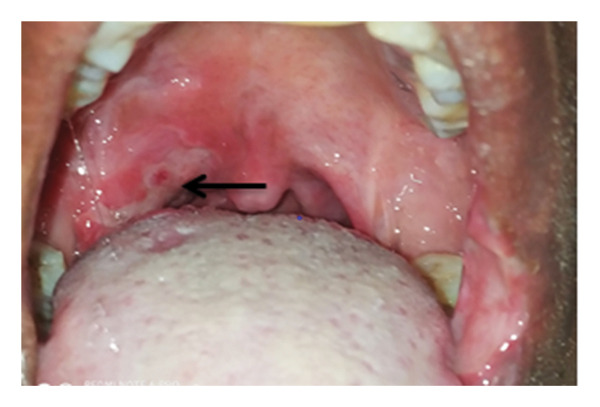
Swollen palatine tonsil (arrow) with exudates and surrounding hyperemia at presentation.

A 40‐year‐old female patient presented to Dekemhare General Hospital with the chief complaint of painful ulcer on the tip of the tongue, right tonsil, and internal side of the left commissure of the mouth of 2‐year duration, which was associated with painful swallowing and eating, burning sensation which is worsened by physical contact to certain types of foods and drinks, brief relief, and recurrence despite a number of oral antibiotics tried for tonsillitis mouth ulcers in her several visits. Patient claims to have no history of fever, chronic cough, trauma, weight loss, or prior history of TB treatment, contact with chronic cougher, or drug intake or chronic illnesses. On physical examination, the patient was well built and generally stable. In the intraoral examination, there was approximately 2 cm in diameter ulcer with exudate on the left labial mucosa commissure of the mouth, which is characterized by tender, slightly elevated, granulomatous, well‐defined border with whitish central surface. On the right side of the tip of the tongue, there was a small, about 0.5 cm in diameter, ulcerated circular lesion with elevated border (Figure 1). Also, at the rear of the oral cavity right palatine tonsil was swollen, ulcerated, and covered with exudates and the surrounding tissue was hyperemic and swollen (Figure 2). Other physical findings of the neck and chest were normal. While patient was on follow‐up, she was advised on strict oral hygiene and avoiding exacerbating factors. Patient was also tried on triamnisolone acentonide 0.1% (orabase) twice daily for 1 month, doxycycline 100 mg twice daily for 1 month, nystatin suspension four times daily, and prednisolone 20 mg twice daily which was tapered over the next 4 weeks but showed no significant improvement. Considering long standing nonhealing ulcers, differential diagnosis included aphthous ulcers, traumatic ulcers, syphilis, drug reaction, infections (bacterial, viral, or fungal), lichen planus, pemphigus, malignant tumors, hematological disorders, and Wegner’s granulomatosis. They were ruled out based on their clinical presentations, history, and their nonresponse to medications. All these and the epidemiological burden of TB rouse the clinical suspicion for oral TB.

**Figure 3:**
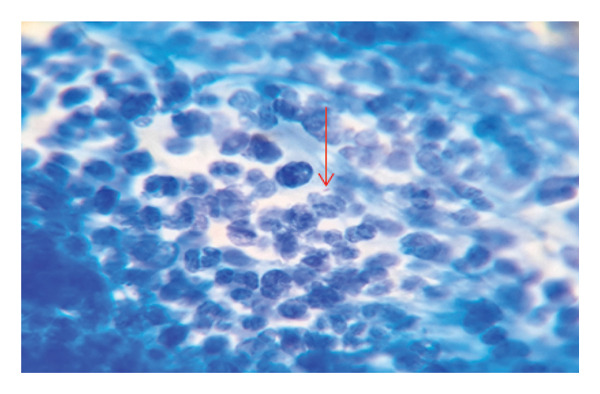
Ziehl–Neelsen method depicting 1 AFB per high power field.

On initial laboratory workup, complete blood count was of normal finding, and erythrocyte‐sedimentation rate (ESR) was 23 mm/hr. Patient was screened for syphilis (VDRL), HIV, and hepatitis C virus, which were negative. The microscopic result showed positive result‐scanty acid‐fast bacilli (AFB), which was 1 AFB per 100 high power field (HPF) after staining with the Ziehl–Neelsen method (Figure 3) from the pus scraped from the tonsil and labial mucosal ulcer. The molecular GeneXpert MTB/RIF test was reactive, which was reported as trace MTB detected and indeterminate rifampicin resistance. Chest X‐ray was done but revealed no significant finding.

**Figure 4:**
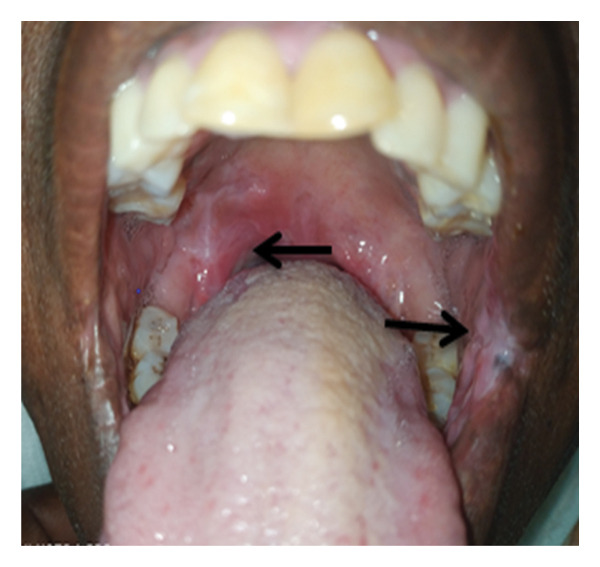
Healing tonsillar and labial mucosa ulcer at 2 weeks after the initiation of anti‐TB.

**Figure 5:**
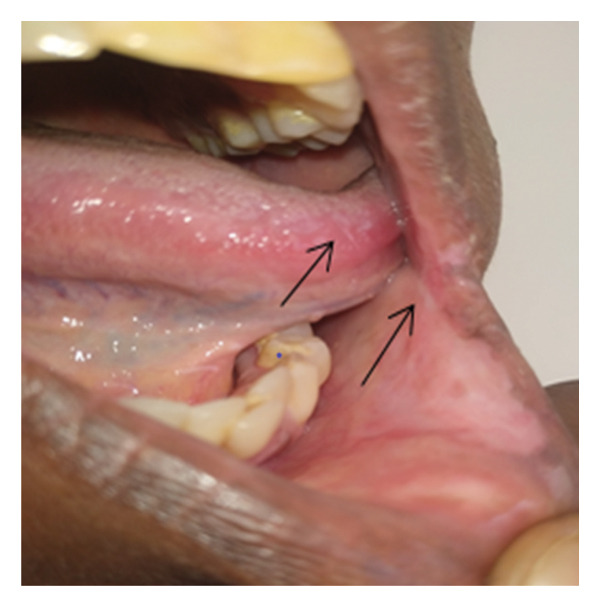
Healed tongue and labial mucosal wound after 2 months of Rx.

**Figure 6:**
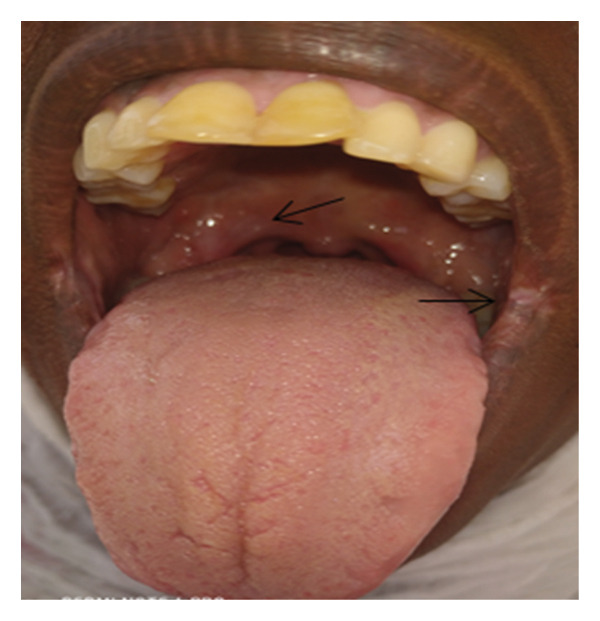
Completely healed lesions after completion of anti‐TB for 6 months.

Patient was initiated on anti‐TB with combination of 4 drugs (isoniazid, rifampicin, pyridoxine, and ethambutol) in the intensive phase for 2 months and then 2 drugs (isoniazid and rifampicin) in the continuation phase for 4 months. Patient’s oral ulcer started responding well after just 2 weeks of anti‐TB initiation (Figure 4) and completely healed after 2 months of treatment (Figure 5). Patient was declared treatment complete after 6 months of treatment as per the guideline (Figure 6).

## 3. Discussion

TB is a global burden; an estimated more than 10 million people continue to fall ill with TB every year [1, 3]. According to Global TB Report‐2023, 7.5 million people were newly diagnosed with TB and caused an estimated 1.30 million deaths in 2022 [1]. TB is one of the major public health problems in Eritrea with an estimated 3700 new TB in 2017 [3]. EPTB refers to a case of TB involving organs other than the lungs and accounts for approximately 20% of the TB cases [7–9]. Symptoms of EPTB are constitutional symptoms like, fever, night sweats and weight loss and symptoms and signs which are specific to the organ involved [3]. TB lymph adenitis is the most common sites of TB [7, 8, 10], other sites of EPTB are pleura, skeletal system, CNS, abdomen, genitourinary, and pericardium, and miliary TB is also common EPTB [4, 8, 10].

Oral cavity TB is usually secondary, which is commonly accompanied by initial pulmonary lesions or other sites of infection. Primary oral TB is a very rare occurrence [4, 11]. Primary lesions develop when TB bacilli are directly inoculated from exogenous source into the oral tissues or any vulnerable potential site of a person who has not acquired immunity to the disease. These frequently involve gingiva, tooth extraction sockets, and buccal folds. In contrast, secondary lesions of oral tissues can result from spreading either via self‐inoculation from or through blood/lymph or from auto inoculation by infected sputum and direct extensions from neighboring structures [12]. The incidence of oral lesions varies by sites, most common sites are tongue, gum, and palate [13], more than half affects the tongue [14], palatine tonsil accounts for < 0.5% of the cases [8], and other sites include the lip, cheek, uvula, and alveolar mucosa [13]. The least affected sites are the upper lip and soft palate [15]. The rarity of primary oral TB is considered to be due to protective action of saliva, salivary enzymes, tissue antibodies, oral saprophytes, stratified squamous thickness of mucosa, and inherent resistance of tonsil to tuberculous infection [10, 16]. Majority (about 93%) of the oral lesions are ulcers; however, patches, indurated soft‐tissue lesions, nodules, fissures, plaques, granulomas, or verrucous proliferations may be seen [14]. The presented case has ulceration on the tongue, palatine tonsil, and labial mucosa. Breaching of the epithelium is important for primary oral TB to occur in most cases [13, 17]. Poor dental hygiene, trauma, dental extraction, periodontitis, and leucoplakia are usually the predisposing factors [8, 14, 18]. The case reported here is immunocompetent, and no predisposing factor was mentioned save poor dental hygiene.

Diagnosis of oral TB like other forms of EPTB present with its challenges due to difficulties of obtaining samples [9] and paucibacillary tissues, thus decreasing the yield of diagnostic tests [9, 13]. The yield from conventional AFB smear on Ziehl–Neelsen staining is very low, ranges 0%–40% [9], even with histopathological examination the yield is only 27%–60% [13]. Culture positive results vary from 30% up to 80%, but it usually takes 2–8 weeks [9]. Biopsy remains the better investigative modalities from which other tests can be done to increase the yields [9, 13]. GeneXpert MTB/RIF assay has an overall sensitivity of 83.1% and a pooled specificity of 98.7% for the diagnosis of EPTB [9] and can detect as few as 10 AFB [9]. In the reported case, the diagnosis was reached by Ziehl–Neelsen staining from pus scrubbed from the ulcer (*scanty-1 AFB per 100 HPF* was found) and GeneXpert MTB/RIF test (*positive result showed trace MTB detected and indeterminate rifampicin resistance*). These results show the rarity of the mycobacterium in such cases. Chest X‐ray and sputum AFB stains were done to rule out pulmonary involvement but revealed no positive result.

Primary oral TB cases respond well to standard anti‐TB treatment [4, 8, 17] which is given under directly observed treatment short (DOTS) course strategy recommended by the World Health Organization (WHO) [3, 5]. Our case was treated for 6 months to avoid any chances of recurrence and resistance though the wound completely healed in 2 months.

It is important to highlight that TB is a contagious disease and dental procedures can be traumatic. It is reasonable to maintain an effective and tight infection control programs in dental clinics to minimize nosocomial infections and occupational hazards. The use of personal protective equipment, proper sterilization protocols, and universal precautions must be strictly enforced [5, 14]. Besides, symptomatic patients should be isolated and promptly referred for medical care. All elective dental procedures should be deferred until the patient is noninfectious, and urgent dental treatment should be carried out in airborne infection isolation facilities [5].

## 4. Conclusion

TB burden has been increasing in the past years, especially in developing countries. TB does not spare any part of the body. Long standing and nonresponding ulcers to antibiotics in any case and any site should always raise the possibility of TB as a differential diagnosis. Like other forms of EPTB, primary oral TB presents with its diagnostic challenges, so meticulous examination and consideration are vital for early detection and initiation of anti‐TB. Likewise, to prompt early identification and intervention, oral healthcare providers should be aware of the various clinical presentations of oral TB. All cases of oral TB respond well to standard WHO‐based‐guideline treatments. We presented this case as the presence of primary oral TB in an immunocompetent case without pulmonary TB is a very unusual presentation.

**Nomenclature**
TB: Tuberculosis
EPTB: Extrapulmonary tuberculosis
AFB: Acid fast bacilli
VDRL: Venereal Disease Research Laboratory
HPF: High power field
MTB/RIF: *Mycobacterium tuberculosis*/Rifampicin
HIV/AIDS: Human immunodeficiency virus/acquired immunodeficiency syndrome
WHO: World Health Organization
DOTS: Directly Observed Treatment Short

## Author Contributions

Yosief Yemane: conceptualization, patient management, supervision, data collection, and writing–original draft.

Hailemichael Ghebremariam: data collection, literature review, and writing–review and editing.

Teklezghi Ainealem: patient care, follow‐up, resources, and writing–review.

Haben Daniel: dental examination, imaging contribution, and clinical validation.

Natnael Ghebregziabher: methodology, patient management, and writing–review and editing.

## Funding

No funding was received for this manuscript.

## Disclosure

All authors read and approved the final manuscript.

## Ethics Statement

Written informed consent was obtained from the patient for participation in this study. In addition, specific consent to publish the clinical details and identifiable intraoral images was obtained.

## Conflicts of Interest

The authors declare no conflicts of interest.

## Patient Perspective

Even though the patient was with painful oral lesions for 2 years and finally diagnosed with oral TB, she was delightful to take the long‐duration anti‐TB. The patient experienced no complications while on medications and had convenient healing. The patient was properly followed for 6 months. Eventually, the patient was satisfied with the treatment protocol and experienced no recurrence of oral ulcers.

## Supporting Information

The CARE checklist has been completed by the authors for this case report, attached as online supporting information.

## Supporting information


**Supporting Information** Additional supporting information can be found online in the Supporting Information section.

## Data Availability

Data sharing is not applicable to this article as no datasets were generated or analyzed during the current study.
