# Supplementary material for: Primary Oral Tuberculosis of Tongue, Tonsil, and Labial Mucosa: A Rare Case Report
Source: Case Rep Med. 2026 Feb 17;2026:6678465. doi: 10.1155/carm/6678465 (PMC12910516; doi:10.1155/carm/6678465)
Supplement: Supplementary file 1 — Supporting Information Additional supporting information can be found online in the Supporting Information section. [file CARM-2026-6678465-s001.docx]

CARE Checklist for case Report

**(Primary Oral Tuberculosis of Tongue, Tonsil, and Labial Mucosa: A Rare Case Report)**

| Section/ Topic | | | Checklist Item | | Reported in Manuscript | |
| --- | --- | --- | --- | --- | --- | --- |
| Title | 1. The Words ’Case Report’ should appear in the Title | | Yes-: Primary Oral Tuberculosis Of Tongue, Tonsil And Labial Mucosa: A Case Report | |  |  |
| Key words | 2. 6 key words | | Yes- Extra-pulmonary Tuberculosis, Ziehl-neelsen, gene-xpert, tongue, tonsil, Case Report | |  |  |
| Abstract | 3a. Introduction: What is unique | | Yes- Mentions rarity of Oral and Diagnostic Challenges | |  |  |
|  | 3b. Main symptoms and clinical findings | | Yes- Painful Oral Ulcers, dysphagia, Swallowing difficulties | |  |  |
|  | 3c. Diagnosis and intervention | | Yes- Ziehl-Nelson staining, Gene-xpert | |  |  |
|  | 3d. Outcomes | | Yes- Complete healing in 2 months, treatment completed in 6months | |  |  |
|  | 3e. Conclusion | | Yes- Importance of High Suspicion for Diagnosis | |  |  |
| Introduction | 4. Brief background of conditions & significance | | Yes- epidemiology, rarity of oral TB, relevance. | |  |  |
| Patient information | 5a. Identified demographic info (age, sex, ethnicity, occupation) | | Yes -40-years-old female patient (other personal details not revealed) | |  |  |
|  | 5b. Chief complaints/ symptoms | | Yes- painful ulcers, dysphagia, burning sensation. | |  |  |
|  | 5c. Medical, family, psychosocial history | | Partial- No history of TB, fever, chronic cough, trauma, weight loss, contact with chronic cougher, drug intake or chronic illness | |  |  |
| Clinical findings | 6. Relevant physical exam findings | | Yes –tongue ulcer, tonsil lesion, labial mucosa ulcer described | |  |  |
| Diagnostic Assessment | 8a.Diagnostics methods (lab, imaging, etc) | | Yes- CBC, ESR, VDRL, HIV- test, hepatitis c virus antibody test, Ziehl-Neelsen, Gene-xpert, chest x-ray. | |  |  |
|  | 8b. Diagnostic reasoning including differential diagnosis | | Yes- Aphthous ulcers, traumatic ulcers, syphilis, drug reaction, lichen planus, pemphigus, malignant tumors, infections (bacterial, viral, fungal), hematological disorders, and Wegner’s granulomatosis. | |  |  |
|  | 8c. Diagnostic challenge | | Yes- rarity, Pauci-bacillary nature, low yield | |  |  |
| Therapeutic Intervention | 9a. Type of Intervention | | Yes- WHO standard anti-TB regimen | |  |  |
|  | 9b. Administration and Dosage | | Yes- 4-Drug regimen Intensive Phase (2 months) And 2-drug regimen Continuation Phase (4 months) | |  |  |
|  | 9c. Changes in intervention | | Not- Applicable- No changes required | |  |  |
| Follow-up and outcomes | 10a. clinician- and patient-assessed outcomes | | Yes-Ulcer healing, stable condition | |  |  |
|  | 10b. Important follow-up results | | Yes- response 2 weeks, full healing after 2 months. | |  |  |
| Discussion | 11a. Strengths/Limitations of the case | | Yes- rarity, diagnostic challenge, but limited diagnostic modalities. | |  |  |
|  | 11b. Comparison with Literature | | Yes- referenced several Oral Tb case reports and reviews | |  |  |
|  | 11c. Conclusion (take-away lesson) | | Yes-Importance of Considering TB in chronic non-healing oral ulcers. –Oral health care providers should be aware of different clinical presentation of oral TB. | |  |  |
| Patient’s Perspective | 12. Patient own experience | | Yes- Patient was satisfied with the treatment protocol and experienced no recurrence of oral ulcers. | |  |  |
| Author’s contribution | 13. Statement detailing how each author contributed | | Yes- author’s contribution mentioned. | |  |  |
| Informed consent | 14. Written informed consent and consent to publish obtained | | Yes- Explicitly mentioned. | |  |  |
